# Adaptive Extended Kalman Filter with Correntropy Loss for Robust Power System State Estimation

**DOI:** 10.3390/e21030293

**Published:** 2019-03-18

**Authors:** Zhiyu Zhang, Jinzhe Qiu, Wentao Ma

**Affiliations:** School of Automation and Information Engineering, Xi’an University of Technology, Xi’an 710048, China

**Keywords:** correntropy loss, extended Kalman filter, adaptive update mechanism, power system robust state estimation, non-Gaussian noises

## Abstract

Monitoring the current operation status of the power system plays an essential role in the enhancement of the power grid for future requirements. Therefore, the real-time state estimation (SE) of the power system has been of widely-held concern. The Kalman filter is an outstanding method for the SE, and the noise in the system is generally assumed to be Gaussian noise. In the actual power system however, these measurements are usually disturbed by non-Gaussian noises in practice. Furthermore, it is hard to get the statistics of the state noise and measurement noise. As a result, a novel adaptive extended Kalman filter with correntropy loss is proposed and applied for power system SE in this paper. Firstly, correntropy is used to improve the robustness of the EKF algorithm in the presence of non-Gaussian noises and outliers. In addition, an adaptive update mechanism of the covariance matrixes of the measurement and process noises is introduced into the EKF with correntropy loss to enhance the accuracy of the algorithm. Extensive simulations are carried out on IEEE 14-bus and IEEE 30-bus test systems to verify the feasibility and robustness of the proposed algorithm.

## 1. Introduction

The power system state estimation (SE) is the foundation and core of the energy management system, and it is indispensable for power system safety, reliability, quality and economic operation [1]. SE is usually divided into static state estimation (SSE) and forecasting-aided state estimation (FASE), FASE is also called dynamic state estimation in some studies [2]. SSE can grasp the real-time operating state of the power system, and FASE can predict the operating trend of the system. The power system is a quasi-steady state system and the state information of buses will change with the change of loads. The SSE cannot consider the impact of the change of system loads on system status information, while the FASE with the function of analyzing and predicting the operation trend of the system is required. Therefore, research on power system forecasting-aided state estimation (PSFASE) is of great significance [3,4,5].

At present, the achievements of FASE are mainly based on different Kalman filters and their modifications. The original Kalman filtering algorithm can only solve linear problems, and it loses its advantage when facing complex nonlinear systems like a power system. Subsequently, an extended Kalman filter (EKF) was proposed in [6], which can linearize the nonlinear measurement function by Taylor series and can be applied in the nonlinear system. In [7], EKF has been used to solve the PSFASE. Considering the adaptability of the algorithm is poor when the power system sudden load changes, an adaptive EKF was proposed in [8]. To enhance the robustness of the algorithm when bad data exists in the measurement data, the robust EKF was proposed in [9]. A new robust generalized maximum-likelihood-type EKF is developed in [10] which can bound the influence of the disturbances. To avoid first-order approximation errors of the EKF, which may be large under strong nonlinearities of the model, the iterated EKF (IEKF) [11] has been proposed as an alternative method. The IEKF iteratively linearizes the functions of nonlinear system to compensate for the higher order terms. To improve the stability of algorithm when the system model involves uncertainty, the H∞ EKF was proposed in [12] which quotes the robust control theory. To summarize, the EKF and its modifications make a great breakthrough in the optimization of the nonlinear system and widely applied for the PSFASE due to its simplicity, high computational efficiency and superior performance in highly nonlinear systems. 

Although EKF can solve the nonlinear problem, these methods ignore the influence of higher order terms in the process of linearization and increase the computational complexity because it needs to calculate Jacobian matrix. For resolving the above challenge, the unscented Kalman filter (UKF) was developed in [13] to approximate the nonlinear distribution by sampling method to solve the nonlinear problem. In [14], a UKF based power system dynamic state estimation was proposed. However, all these approaches mentioned above have suffered from several important defects, limiting them from being adopted for PSFASE. To be specific, they cannot handle: 1) the non-Gaussian process and observation noises of the system nonlinear dynamic models, and 2) the unknown noise covariance matrices. In the actual power grids, the non-Gaussian process and observation noises are ubiquitous and hence the inappropriate noise covariance matrices will lead to imprecise estimation results. 

The traditional EKF and UKF methods are derived based on mean square error (MSE) which is optimal under Gaussian assumption. Therefore, the SE methods based on the original EKF and UKF will show un-robustness when the system suffers from the non-Gaussian noises, that is, the state cannot be estimated correctly. To overcome the influence of non-Gaussian noise, a novel maximum correntropy criteria (MCC) [15,16,17,18,19] has been developed in an information theoretic learning (ITL) methodology for non-Gaussian signal processing. At present, a novel robust EKF based on the MCC (called MCC-EKF) was developed in which the MSE was substituted by the MCC to solve estimation issues in non-Gaussian noise environments [20,21,22]. In this paper, the MCC-EKF is firstly used to design a robust SE method to suppress the interference of non-Gaussian noise. In addition, a novel adaptive update mechanism of the predicted error covariance matrix and measurement noise covariance matrix is introduced into the MCC-EKF, and an adaptive MCC-EKF (called AMCC-EKF) method is proposed to enhance the tracking ability of the original MCC-EKF. The performance of the proposed AMCC-EKF, MCC-EKF, UKF and EKF for PSFASE are tested in this paper. Experimental results illustrate that the proposed algorithm performs better than other algorithms with respect to the estimation accuracy under the non-Gaussian circumstances. 

The remainder of this paper is organized as follows. In Section 2 we briefly review the correntropy and extended Kalman filter. In Section 3 we derived the proposed AMCC-EKF algorithm and in Section 4 the proposed algorithm is applied to PSFASE. Section 5 gives the simulation results. Finally, Section 6 concludes with a summary of the main findings of this paper.

## 2. Preliminaries

### 2.1. Maximum Correntropy Criteria

Given two random variables X and Y, the correntropy is defined as

(1) $$V_{\sigma }(X,Y)=E[G_{\sigma }(X-Y)]=\iint_{x,y}G_{\sigma }(x-y)dF_{X,Y}(x,y)$$

where E[⋅] denotes the expectation operator, $F_{X,Y}(x,y)$ is the joint distribution function of X and Y, and $G_{\sigma }(\cdot )$ represents a shift-invariant Mercer kernel, with bandwidth σ. In this paper, we use the following Gaussian kernel

(2) $$G_{\sigma }\left(x-y\right)=\exp\left(-\frac{{\left\|x-y\right\|}^{2}}{2\sigma^{2}}\right)$$

**Figure 1:**
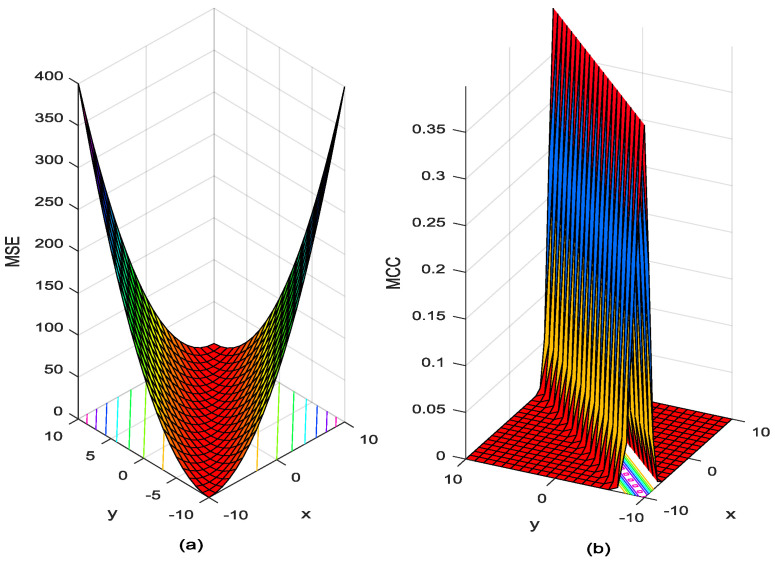
Performance surface of MSE and Correntropy: (**a**) MSE (**b**) Correntropy.

Correntropy can be used as a cost function to develop novel robust adaptive filtering algorithms [23,24,25]. In practice, only finite samples of the variables X and Y are given, and the joint probability density function is unknown in general. Hence the sample mean estimator of corrrentropy is used as

(3) $${\hat{V}}_{\sigma }(X,Y)=\frac{1}{N}\sum_{i=1}^{N}G_{\sigma }(x_{i}-y_{i})$$

where N is the number of samples. And the performance surface of the maximum square mean (MSE) and correntropy are shown in Figure 1. It is can be seen that correntropy is local whereas MSE is global. By global, we mean that all the samples in the joint space will contribute appreciably to the value of the similarity measure while the locality of correntropy means that the value is primarily dictated by the kernel function along the x=y line. Therefore, correntropy of the error can be used as a robust cost function for adaptive systems training, which will be called the MCC. MCC has the advantage that it is a local criterion of similarity and it should be very useful for cases when the measurement noise is nonzero mean, non-Gaussian, with large outliers [19].

### 2.2. Review of Extended Kalman Filter

The extended Kalman filter, a derivation of Kalman filter, is developed to resolve the problem of nonlinear system by Taylor series. Consider a nonlinear system by the nonlinear state and linear measurement functions:

(4) $$x_{k}=f\left(x_{k-1}\right)+w_{k-1}$$

(5) $$y_{k}=H_{k}x_{k}+v_{k}$$

where $x_{k}\in R^{n}$ denotes a n-dimensional state vector at time step k, $y_{k}\in R^{m}$ denotes a m-dimensional measurement vector at time step k, f(⋅) represents the vector-valued function and $H_{k}$ stands for the observation matrix, $w_{k}$ and $v_{k}$ are process and measurement noises respectively, which are generally assumed to be uncorrelated with zero mean and covariance matrices. For EKF, the measurement noise $v_{k}$ is also assumed to be a zero mean Gaussian white noise sequence while the MCC-EKF avoids the Gaussian assumption of $v_{k}$.

(6) $$E\left[w_{k-1}w^{T}{}_{k-1}\right]=Q_{k}$$

(7) $$E\left[v_{k}v^{T}{}_{k}\right]=R_{k}$$

In general, extended Kalman filter includes the following two steps:

(1) predict: the prior mean and covariance matrix are given by:

(8) $${\hat{x}}_{k|k-1}=f\left({\hat{x}}_{k-1|k-1}\right)$$

(9) $$P_{k|k-1}=F_{k-1}P_{k-1|k-1}F^{T}{}_{k-1}+Q_{k-1}$$

where ${\hat{x}}_{k|k-1}$ denotes the predicted state vector at time (*k*−1). $F_{k-1}$ denotes a Jacobian matrix of f(⋅), and is described as $F_{k-1}=\frac{\partial f\left({\hat{x}}_{k-1|k-1}\right)}{\partial x_{k}}$.

(2) update: the gain matrix of extended Kalman filter can be obtained as

(10) $$K_{k}=P_{k|k-1}H_{k}^{T}{\left(H_{k}P_{k|k-1}H_{k}^{T}+R_{k}\right)}^{-1}$$

the posterior state is equal to the prior state plus the innovation weighted by the gain matrix of extended Kalman filter

(11) $${\hat{x}}_{k|k}={\hat{x}}_{k|k-1}+K_{k}\left(y_{k}-H_{k}{\hat{x}}_{k|k-1}\right)$$

Additionally, the iterative formula for the posterior covariance is as follows

(12) $$P_{k|k}=\left(I-K_{k}H_{k}\right)P_{k|k-1}{\left(I-K_{k}H_{k}\right)}^{T}+K_{k}R_{k}K_{k}^{T}$$

## 3. Adaptive Extended Kalman Filter With Correntropy Loss

### 3.1. Extended Kalman Filter with Correntropy Loss

The traditional EKF based on MSE loss and it is not robust when the system noise is non-Gaussian. To overcome the influence of non-Gaussian noise, a novel maximum correntropy criteria (MCC) has been developed in ITL. In [15], the MCC-EKF was developed by using the MCC to replace the MSE, which may perform much better in non-Gaussian noise environments. The main reason is that the correntropy contains second and higher order moments of the error, that is, the correntropy in ITL involves all even moments of the error which can be obtained by Taylor expansion [15].

For the nonlinear model given by (4) (5) and expression (8) (9), we can obtain

(13) $$\left[\begin{array}{c} {\hat{x}}_{k|k-1}\\ y_{k} \end{array}\right]=\left[\begin{array}{c} I\\ H_{k} \end{array}\right]x_{k}+q_{k}$$

We assumed that the state vector $x_{k}$ and process noise $w_{k}$ and measurement noise $v_{k}$ are non-zero correlation respectively.

Where I is the n×n identity matrix, and $q_{k}$ can express as

(14) $$q_{k}=\left[\begin{array}{c} -\left(x_{k}-{\hat{x}}_{k|k-1}\right)\\ v_{k} \end{array}\right]$$

With

(15) $$\begin{array}{ll} E\left[q_{k}q_{k}^{T}\right] & =\left[\begin{array}{cc} P_{k|k-1} & 0\\ 0 & R_{k} \end{array}\right]\\ & =\left[\begin{array}{cc} B_{p,}{}_{k|k-1}B_{p,}^{T}{}_{k|k-1} & 0\\ 0 & B_{r}{}_{,k}B_{r,}^{T}{}_{k} \end{array}\right]\\ & =B_{k}B_{k}^{T} \end{array}$$

where $B_{k}$ can be computed by Cholesky decomposition of $E\left[q_{k}q_{k}^{T}\right]$. Left multiplying both sides of (13) by $B^{-1}{}_{k}$, we have

(16) $$D_{k}=W_{k}x_{k}+e_{k}$$

where $D_{k}=B_{k}^{-1}\left[\begin{array}{c} {\hat{x}}_{k|k-1}\\ y_{k} \end{array}\right]$, $w_{k}=B_{k}^{-1}\left[\begin{array}{c} I\\ H_{k} \end{array}\right]$, $e_{k}=B_{k}^{-1}v_{k}$. Since $E\left[e_{k}e_{k}^{T}\right]=I$, the residual error $e_{k}$ is white.

Then, we define the following cost function $J_{L}\left(x_{k}\right)$ based on MCC

(17) $$J_{L}\left(x_{k}\right)=\frac{1}{L}\sum_{i=1}^{L}G_{\sigma }\left(d_{i,k}-w_{i,k}x_{k}\right)$$

where $d_{i,k}$ is the *i*-th element of $D_{k}$, $w_{i,k}$ is the *i*-th row of $W_{k}$, and L=m+n is the dimension of $D_{k}$.

Under the MCC, the optimal estimate of $x_{k}$ is

(18) $${\hat{x}}_{k}=\arg\underset{x_{k}}{\max}J_{L}\left(x_{k}\right)=\arg\underset{x_{k}}{\max}\sum_{i=1}^{L}G_{\sigma }\left(e_{i,k}\right)$$

where $e_{i,k}$ is the *i*-th element of $e_{k}$ and

(19) $$e_{i,k}=d_{i,k}-w_{i,k}x_{k}$$

Hence, the optimal solution can be obtained by solving

(20) $$\frac{\partial J_{L}\left(x_{k}\right)}{\partial x_{k}}=0$$

It follows easily that

(21) $$x_{k}={\left(\sum_{i=1}^{L}\left[G_{\sigma }\left(e_{i,k}\right)w_{i,k}^{T}w_{i,k}\right]\right)}^{-1}\times \left(\sum_{i=1}^{L}\left[G_{\sigma }\left(e_{i,k}\right)w_{i,k}^{T}d_{i,k}\right]\right)$$

Since $e_{i,k}=d_{i,k}-w_{i,k}x_{k}$, the formula (21) is a of $x_{k}$ and can be rewritten as

(22) $$x_{k}=g\left(x_{k}\right)$$

A fixed-point iterative algorithm can be expressed as

(23) $${\hat{x}}_{k,t+1}=g\left({\hat{x}}_{k,t}\right)$$

where ${\hat{x}}_{k,t}$ denotes the state ${\hat{x}}_{k}$ at the fixed-point iteration *t*

The Equation (21) can also be expressed as

(24) $$x_{k}={\left(W_{k}^{T}C_{k}W_{k}\right)}^{-1}\times W_{k}^{T}C_{k}D_{k}$$

where $C_{k}=\left[\begin{array}{cc} C_{x,k} & 0\\ 0 & C_{y,k} \end{array}\right]$, with

(25) $$C_{x,k}=diag\left(G_{\sigma }\left(e_{1,k}\right),\ldots ,G_{\sigma }\left(e_{n,k}\right)\right)$$

(26) $$C_{y,k}=diag\left(G_{\sigma }\left(e_{n+1,k}\right),\ldots ,G_{\sigma }\left(e_{n+m,k}\right)\right)$$

Based on formula (24), we can obtain a recursive formula [20]

(27) $$x_{k}={\hat{x}}_{k|k-1}+{\bar{K}}_{k}\left(y_{k}-H_{k}{\hat{x}}_{k|k-1}\right)$$

where

(28) $${\bar{K}}_{k}={\bar{P}}_{k|k-1}H_{k}^{T}{\left(H_{k}{\bar{P}}_{k|k-1}H_{k}^{T}+{\bar{R}}_{k}\right)}^{-1}$$

(29) $${\bar{P}}_{k|k-1}=B_{p,k|k-1}^{}C_{x,k}^{-1}B_{p,k|k-1}^{T}$$

(30) $${\bar{R}}_{k}=B_{r,k}C_{y,k}^{-1}B_{r,k}^{T}$$

With the above derivations, the optimal state variable of MCC-EKF algorithm can be obtained by the formula (25)–(30).

### 3.2. Adaptive Extended Kalman Filter with Correntropy Loss

The traditional EKF algorithm is usually used under the condition that the statistical characteristics of state noise and measurement noise of the system are known, but it is unknown in the actual situation. Therefore, the filtering divergence and inaccurate estimation results may be generated by the influence of the uncertain factors. Similarity to the EKF, The MCC-EKF algorithm will still suffer from this problem. In order to improve the accuracy of MCC-EKF algorithm, a covariance matrix adaptive mechanism is introduced into the MCC-EKF algorithm to continuously estimate and modify the filter noise statistical characteristics online, and we call the novel algorithm adaptive MCC-EKF algorithm which can improve the filtering accuracy while filtering by using the information of observation data. Thus, the optimal value of the estimated state is obtained [26,27]. Now, the covariance matrix adaptive update mechanism is given.

We define the new information $d_{k}$ that is the error with the actual observation value $y_{k}$ and the predicted observation value ${\hat{y}}_{k|k-1}$ at time *k* as

(31) $$d_{k}=y_{k}-{\hat{y}}_{k|k-1}=H_{k}x_{k}+v_{k}-H_{k}{\hat{x}}_{k|k-1}=H_{k}{\overset{⌢}{x}}_{k|k-1}+v_{k}$$

where ${\overset{⌢}{x}}_{k|k-1}=x_{k}-{\hat{x}}_{k|k-1}$ denotes one-step prediction error.

Then, according to the windowing estimation method, the real-time estimation variance of the new information $d_{k}$ is

(32) $${\hat{C}}_{d_{k}}=\{\begin{array}{c} \frac{k-1}{k}{\hat{C}}_{d_{k-1}}+\frac{1}{k}d_{k}d_{k}^{T},k\leq W\\ \frac{1}{W}\sum_{i=k-W+1}^{k}d_{i}d_{i}^{T},k>W \end{array}$$

where W is the size of the moving window. Since $v_{k}$ and ${\hat{x}}_{k|k-1}$ are uncorrelated, we have

(33) $${\hat{C}}_{d_{k}}=E\left(d_{k}d_{k}^{T}\right)=H_{k}P_{k|k-1}H_{k}^{T}+R_{k}$$

The residual $r_{k}$ at time *k* is defined as the error with the actual observed value $y_{k}$ and the estimated observed value ${\hat{y}}_{k}$

(34) $$\begin{array}{ll} r_{k} & =y_{k}-{\hat{y}}_{k}\\ & =y_{k}-H_{k}\left[{\hat{x}}_{k|k-1}+K_{k}\left(y_{k}-H_{k}{\hat{x}}_{k|k-1}\right)\right]\\ & =\left(I-H_{k}K_{k}\right)d_{k}\\ & =\left(I-\frac{H_{k}P_{k|k-1}H_{k}^{T}}{H_{k}P_{k|k-1}H_{k}^{T}+R_{k}}\right)d_{k}\\ & =R_{k}C_{d_{k}}^{-1}d_{k} \end{array}$$

The variance of the residual $r_{k}$ is defined as

(35) $$C_{r_{k}}=E\left(r_{k}r_{k}^{T}\right)=R_{k}C_{d_{k}}^{-1}C_{d_{k}}C_{d_{k}}^{-1}R_{k}=R_{k}C_{d_{k}}^{-1}R_{k}$$

According to Kalman filtering principle [26,27,28,29], the filtering gain is

(36) $$K_{k}=P_{k|k-1}H_{k}^{T}C_{d_{k}}^{-1}=P_{k}H_{k}^{T}R_{k}^{-1}$$

Left multiplying both sides of Equation (36) by $H_{k}$, and substitute into Equation (33), we can obtain

(37) $$\begin{array}{ll} H_{k}P_{k}H_{k}^{T}R_{k}^{-1} & =H_{k}P_{k|k-1}H_{k}^{T}C_{d_{k}}^{-1}\\ & =\left(C_{d_{k}}-R_{k}\right)C_{d_{k}}^{-1}\\ & =I-R_{k}C_{d_{k}}^{-1} \end{array}$$

Right multiplying both sides of Equation (37) by $R_{k}$, we have

(38) $$H_{k}P_{k}H_{k}^{T}=R_{k}-R_{k}C_{d_{k}}^{-1}R_{k}$$

Combining (35) and (38), we can get the ${\hat{R}}_{k}$ as follows

(39) $${\hat{R}}_{k}={\hat{C}}_{r_{k}}+H_{k}P_{k}H_{k}^{T}$$

Similarly, the new information $d_{k}$ can be used to estimate the variance matrix Q of the state noise, Right multiplying both sides of Equation (36) by $C_{d_{k}}K_{k}^{T}$, we have

(40) $$K_{k}C_{d_{k}}K_{k}^{T}=P_{k|k-1}H_{k}^{T}C_{d_{k}}^{-1}C_{d_{k}}K_{k}^{T}=P_{k|k-1}H_{k}^{T}K_{k}^{T}$$

Transposing both sides of formula (22) and applying Kalman filtering principle, we can obtain

(41) $$K_{k}C_{d_{k}}K_{k}^{T}=P_{k|k-1}-P_{k}=F_{k,k-1}P_{k}F_{k,k-1}^{T}+Q_{k-1}-P_{k}$$

To keep Q semi-positive, the approximate estimate of $Q_{k-1}$ can be expressed as

(42) $${\hat{Q}}_{k-1}=K_{k}{\hat{C}}_{d_{k}}K_{k}^{T}$$

## 4. Adaptive Extended Kalman Filter With Correntropy Loss for PSDSE

### 4.1. Power System Dynamic Model

The power system is a nonlinear and complex system. For the FASE of power system, dynamic equation and measurement equation can be expressed in the following form

(43) $$x_{k}=f\left(x_{k-1}\right)+w_{k-1}$$

(44) $$y_{k}=h\left(x_{k}\right)+v_{k}$$

where $x_{k}$ denotes the state vector consisting of magnitudes and angles of nodal voltage. The measurement vector $y_{k}$ comprises of voltage magnitude measurements, real power injection measurements, reactive power injection measurements, real power flow measurements, reactive power flow measurements. The noise $w_{k}$ and $v_{k}$ are usually assumed to be Gaussian noise and independent of each other. Specially, the noise $w_{k}$ is the error of system and the noise $v_{k}$ is the error of measurement. f(⋅) represents the function that relates $x_{k-1}$ to $x_{k}$ and h(⋅) stands for the measurement function that relates $x_{k}$ to $y_{k}$.

In order to determine the dynamic model of power system, it is necessary to identify the model parameters. The Holt’s two-parameter linear exponential smoothing technique [30], also known as linear extrapolation method, is most commonly used to calculate the state transition matrix $F_{k}$, and in this paper we use this method as the dynamic model. It can also be used as a simple short-term load forecasting method. It has the advantages of less storage variables and a faster computation speed, and it is suitable for online calculation. Employing this method, the state transition function f(⋅) is defined as

(45) $${\hat{x}}_{k+1|k}=a_{k}+b_{k}$$

where

(46) $$a_{k}=\alpha_{k}{\hat{x}}_{k|k}+\left(1-\alpha_{k}\right){\hat{x}}_{k+1|k}$$

(47) $$b_{k}=\beta_{k}\left(a_{k}-a_{k-1}\right)+\left(1-\beta_{k}\right)b_{k-1}$$

where both $\alpha_{k}$ and $\beta_{k}$ are parameters lying in the range from 0 to 1, and vectors $a_{k}$ and $b_{k}$ at time k are obtained as (43) and (44). Linearization of the nonlinear model in formula (43) above can be expressed as follows

(48) $${\hat{x}}_{k+1|k}=F_{k}{\hat{x}}_{k|k}+u_{k}+w_{k}$$

where $F_{k}=\frac{\partial f\left(x_{k}\right)}{\partial x_{k}}$, $u_{k}$ is a nonrandom external actor in the expansion. Combining formulae (48) and (45), we have

(49) $$F_{k}=\alpha_{k}\left(1+\beta_{k}\right)$$

(50) $$u_{k}=\left(1+\beta_{k}\right)\left(1-\alpha_{k}\right){\hat{x}}_{k+1|k}-\beta_{k}a_{k-1}+\left(1-\beta_{k}\right)b_{k-1}$$

The composition of the measurement vector $y_{k}$ changes with the measurement method of the power system. This paper studies the data measured by SCADA system, so h(x) is a nonlinear function. For bus i, the relationship between the measurement and the state vector as follows [31]

(51) $$P_{i}=\sum_{j=1}^{N}\left|V_{i}\right|\left|V_{j}\right|\left(G_{ij}\cos\theta_{ij}+B_{ij}\sin\theta_{ij}\right)$$

(52) $$Q_{i}=\sum_{j=1}^{N}\left|V_{i}\right|\left|V_{j}\right|\left(G_{ij}\sin\theta_{ij}-B_{ij}\cos\theta_{ij}\right)$$

(53) $$P_{\mathrm{ij}}=V_{i}^{2}G_{ij}-\left|V_{i}\right|\left|V_{j}\right|\left(G_{ij}\cos\theta_{ij}+B_{ij}\sin\theta_{ij}\right)$$

(54) $$Q_{ij}=-V_{i}^{2}\left(B_{gi}+B_{ij}\right)-\left|V_{i}\right|\left|V_{j}\right|\left(G_{ij}\sin\theta_{ij}-B_{ij}\cos\theta_{ij}\right)$$

where $P_{i}$ is the real power injection at bus i, $Q_{i}$ is the reactive power injection at bus i, $P_{ij}$ is the real power flow between buses i and j, $Q_{ij}$ is the reactive power flow between buses i and j, $V_{i}$ is the voltage magnitude at bus i, $G_{ij}$ is the conductance of the line between buses i and j, $B_{ij}$ is the susceptance of the line between buses i and j.

And the Jacobian matrix $H_{k}$ can be expressed as

(55) $$H_{k}={\left[\begin{array}{ccccc} \frac{\partial V_{i}}{\partial \theta } & \frac{\partial P_{i}}{\partial \theta } & \frac{\partial Q_{i}}{\partial \theta } & \frac{\partial P_{ij}}{\partial \theta } & \frac{\partial Q_{ij}}{\partial \theta }\\ \frac{\partial V_{i}}{\partial V} & \frac{\partial P_{i}}{\partial V} & \frac{\partial Q_{i}}{\partial V} & \frac{\partial P_{ij}}{\partial V} & \frac{\partial Q_{ij}}{\partial V} \end{array}\right]}^{T}$$

Combined with the power system network, the specific elements of the Jacobian matrix $H_{k}$ are shown as

(56) $$\{\begin{array}{cc} \frac{\partial V_{i}}{\partial \theta_{i}}=0 & \frac{\partial V_{i}}{\partial \theta_{j}}=0\\ \frac{\partial V_{i}}{\partial V_{i}}=1 & \frac{\partial V_{i}}{\partial V_{j}}=0 \end{array}$$

(57) $$\{\begin{array}{l} \frac{\partial P_{i}}{\partial \theta_{i}}=-V_{i}^{2}B_{ii}-V_{i}\sum_{j=1}^{N}V_{j}\left(G_{ij}\sin\theta_{ij}-B_{ij}\cos\theta_{ij}\right)\\ \frac{\partial P_{i}}{\partial \theta_{j}}=V_{i}V_{j}\left(G_{ij}\sin\theta_{ij}-B_{ij}\cos\theta_{ij}\right)\\ \frac{\partial P_{i}}{\partial V_{i}}=V_{i}G_{ii}+\sum_{j=1}^{N}V_{j}\left(G_{ij}\cos\theta_{ij}+B_{ij}\sin\theta_{ij}\right)\\ \frac{\partial P_{i}}{\partial V_{j}}=V_{i}\left(G_{ij}\cos\theta_{ij}+B_{ij}\sin\theta_{ij}\right) \end{array}$$

(58) $$\{\begin{array}{l} \frac{\partial Q_{i}}{\partial \theta_{i}}=-V_{i}^{2}G_{ii}+V_{i}\sum_{j=1}^{N}V_{j}\left(G_{ij}\sin\theta_{ij}+B_{ij}\cos\theta_{ij}\right)\\ \frac{\partial Q_{i}}{\partial \theta_{j}}=-V_{i}V_{j}\left(G_{ij}\cos\theta_{ij}+B_{ij}\sin\theta_{ij}\right)\\ \frac{\partial Q_{i}}{\partial V_{i}}=-V_{i}B_{ii}+\sum_{j=1}^{N}V_{j}\left(G_{ij}\sin\theta_{ij}-B_{ij}\cos\theta_{ij}\right)\\ \frac{\partial Q_{i}}{\partial V_{j}}=V_{i}\left(G_{ij}\sin\theta_{ij}-B_{ij}\cos\theta_{ij}\right) \end{array}$$

(59) $$\{\begin{array}{l} \frac{\partial P_{\mathrm{ij}}}{\partial \theta_{i}}=-V_{i}V_{j}\left(G_{ij}\sin\theta_{ij}-B_{ij}\cos\theta_{ij}\right)\\ \frac{\partial P_{\mathrm{ij}}}{\partial \theta_{j}}=V_{i}V_{j}\left(G_{ij}\sin\theta_{ij}-B_{ij}\cos\theta_{ij}\right)\\ \frac{\partial P_{ij}}{\partial V_{i}}=2V_{i}G_{ii}-V_{j}\left(G_{ij}\cos\theta_{ij}+B_{ij}\sin\theta_{ij}\right)\\ \frac{\partial P_{ij}}{\partial V_{j}}=-V_{i}\left(G_{ij}\cos\theta_{ij}+B_{ij}\sin\theta_{ij}\right) \end{array}$$

(60) $$\{\begin{array}{l} \frac{\partial Q_{ij}}{\partial \theta_{i}}=V_{i}V_{j}\left(G_{ij}\cos\theta_{ij}+B_{ij}\sin\theta_{ij}\right)\\ \frac{\partial Q_{ij}}{\partial \theta_{j}}=-V_{i}V_{j}\left(G_{ij}\cos\theta_{ij}+B_{ij}\sin\theta_{ij}\right)\\ \frac{\partial Q_{ij}}{\partial V_{i}}=-2V_{i}\left(B_{gi}+B_{ij}\right)-V_{j}\left(G_{ij}\sin\theta_{ij}-B_{ij}\cos\theta_{ij}\right)\\ \frac{\partial Q_{ij}}{\partial V_{j}}=-V_{i}\left(G_{ij}\sin\theta_{ij}-B_{ij}\cos\theta_{ij}\right) \end{array}$$

### 4.2. Adaptive Extended Kalman Filter with Correntropy Loss for Power System Forecasting-Aided State Estimation

Now, we apply this method to PSFASE to solve the problem of non-Gaussian noise and bad data. For *n*-bus power system, there are 2*n*−1 states (contains *n* voltage amplitudes and *n*−1 voltage phase angles) that needs to be estimated. The dynamic model reflects the change law of system state variables with time. The dynamic model of power system in this paper assumes that the changes in the system parameters, such as load variations, are very slow. Then, we give the detail procedure of MCC-EKF algorithm for PSDSE.

1) Select the appropriate initial parameters: a proper kernel bandwidth σ and a small positive ε; Set an initial state value ${\hat{x}}_{0|0}$ and corresponding covariance matrix $P_{0|0}$; Let *k* = 1;
2) Use Equations (8) and (9) to calculate the ${\hat{x}}_{k|k-1}$ and $P_{k|k-1}$, and obtain the $B_{p,k|k-1}$ by Cholesky decomposition;
3) Let *k* = 1 and ${\hat{x}}_{k|k,0}={\hat{x}}_{k|k-1}$, where ${\hat{x}}_{k|k,t}$ stands for the estimated state at the fixed-point iteration *k*;
4) Calculate the state transition function using (45)–(47) and the Jacobian matrix $H_{k}$ using (51)–(60);
5) Get the estimates state ${\hat{x}}_{k|k,t}$ by Equations (61)–(69);
  (61) $${\hat{x}}_{k|k,t}={\hat{x}}_{k|k-1}+{\tilde{K}}_{k}\left(y_{k}-H_{k}{\hat{x}}_{k|k-1}\right)$$
  With
  (62) $${\tilde{K}}_{k}={\tilde{P}}_{k|k-1}H_{k}^{T}{\left(H_{k}{\tilde{P}}_{k|k-1}H_{k}^{T}+{\tilde{R}}_{k}\right)}^{-1}$$
  (63) $${\tilde{P}}_{k|k-1}=B_{p,k|k-1}^{}{\tilde{C}}_{x,k}^{-1}B_{p,k|k-1}^{T}$$
  (64) $${\tilde{R}}_{k}=B_{r,k}{\tilde{C}}_{y,k}^{-1}B_{r,k}^{T}$$
  (65) $${\tilde{C}}_{x,k}=diag\left(G_{\sigma }\left({\tilde{e}}_{1,k}\right),\ldots ,G_{\sigma }\left({\tilde{e}}_{n,k}\right)\right)$$
  (66) $${\tilde{C}}_{y,k}=diag\left(G_{\sigma }\left({\tilde{e}}_{n+1,k}\right),\ldots ,G_{\sigma }\left({\tilde{e}}_{n+m,k}\right)\right)$$
  (67) $${\tilde{e}}_{i,k}=d_{i,k}-w_{i,k}{\hat{x}}_{k|k,t-1}$$
  (68) $${\tilde{R}}_{k}=R_{k}{\left(H_{k}P_{k|k-1}H_{k}^{T}+R_{k}\right)}^{-1}R_{k}+H_{k}P_{k}H_{k}^{T}$$
  (69) $${\tilde{Q}}_{k}=K_{k}\left(H_{k}P_{k|k-1}H_{k}^{T}+R_{k}\right)K_{k}^{T}$$
6) Compare the estimation of the current step and the estimation of the last step. If (70) holds, let ${\hat{x}}_{k|k}={\hat{x}}_{k|k,t}$ and continue to 7). Otherwise, t+1→t, and go back to 5);
  (70) $$\frac{\left||{\hat{x}}_{k|k,t}-{\hat{x}}_{k|k,t-1}|\right|}{\left||{\hat{x}}_{k|k,t-1}|\right|}\leq \varepsilon$$
7) Moreover, the posterior matrix is updated as (71), k+1→k and go back to 2).
  (71) $$P_{k|k}=\left(I-{\tilde{K}}_{k}H_{k}\right)P_{k|k-1}{\left(I-{\tilde{K}}_{k}H_{k}\right)}^{T}+{\tilde{K}}_{k}R_{k}{\tilde{K}}_{k}^{T}$$

## 5. Results

In this section, we perform experiments on the standard IEEE 14-bus and IEEE 30-bus test system to verify the effectiveness and superiority of the proposed algorithm compared with the EKF, UKF, A-EKF and MCC-EKF algorithms. We use the 50 time-sample intervals, which were obtained by running successful load flows under different loading conditions to simulate the slow dynamics of the power system. The variation of loads can be divided into linear and nonlinear variation, among which the linear variation means the whole observed time interval with 50 samples are changes follow a linear trend of 10% and the nonlinear variation denotes the whole observed time interval with 50 samples changes follow a random fluctuation of 5%. The convergence tolerance threshold of all algorithms is 0.001. In addition, all free parameters of the algorithms mentioned above are selected such that each algorithm can achieve its optimal performance.

The mean absolute error (MAE) and root mean square error (RMSE) are utilized to evaluate the performance of each method. In addition, the overall performance is one of the indices for evaluating the performance of the state estimation algorithms, and it can be defined by

(72) $$J_{i}=\frac{\sum_{i=1}^{N}\left|{\hat{y}}_{k}^{i}-{\bar{y}}_{k}^{i}\right|}{\sum_{i=1}^{N}\left|y_{k}^{i}-{\bar{y}}_{k}^{i}\right|}$$

where ${\hat{y}}_{k}^{i}$ is the estimated measurement vector at time index *k* for bus *i*, ${\bar{y}}_{k}^{i}$ denotes the true measurements and $y_{k}^{i}$ represents the measurement vector with noises. The MAE, RMSE and the over performance will be used to verify the available of the proposed method in the following experiments.

### 5.1. Case 1: Gaussian Measurement Noise Environment

In general, the measurement noise is assumed for Gaussian noise, and we first evaluate the proposed algorithm in the normal environment. Loads are changed according to the above specified linear trend. The average overall performance of all algorithms in standard IEEE 30-bus is given in Table 1. From the results, we know that (1) both EKF, UKF and A-EKF algorithms have good performance in this case; (2) the adaptive mechanism can improve the estimation accuracy; (3) the performance of MCC-EKF is slightly better than that of EKF and UKF and the proposed AMCC-EKF performs better than other algorithms which illustrate that on the one hand, the correntropy is robust for the noise, on the other hand, the adaptive mechanism The adaptive mechanism makes the algorithm have stronger tracking performance by updating the predicted error covariance matrix and measurement noise covariance matrix.

### 5.2. Case 2: Gaussian Mixture Measurement Noise Environment

**Figure 2:**
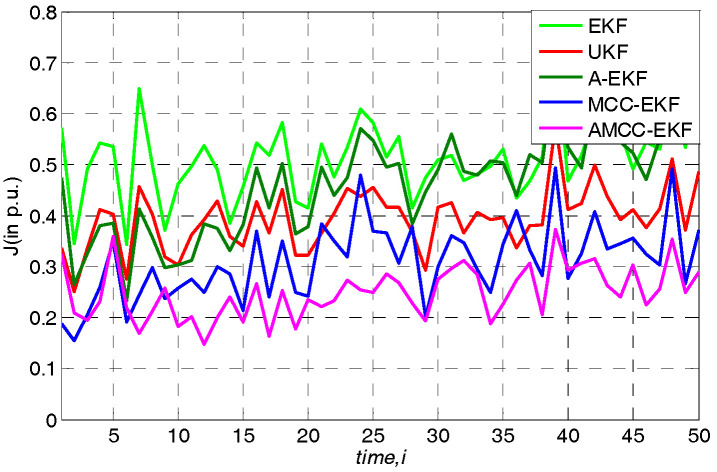
The overall performance of all algorithms in standard IEEE 14-bus under Gaussian mixture measurement noise environment.

**Figure 3:**
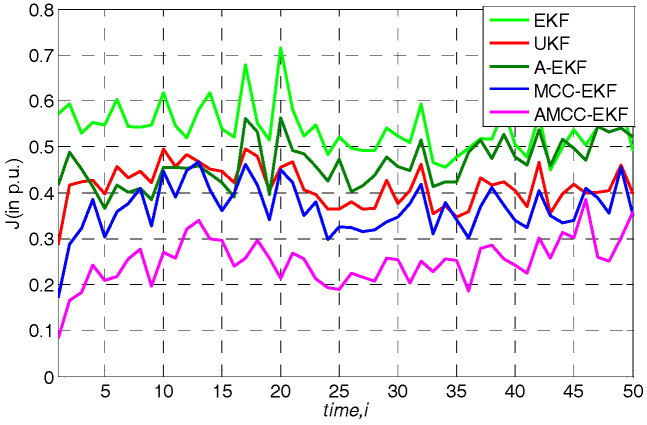
The overall performance of all algorithms in standard IEEE 30-bus under Gaussian mixture measurement noise environment.

In this case, we evaluate the performance of the proposed algorithm under the non-Gaussian measurement noise circumstance. The measurement of noise is modeled by the mixed Gaussian distribution which is defined as

(73) $$\left(1-\theta )N(\mu_{1},\upsilon_{1}^{2})+\theta N(\mu_{2},\upsilon_{2}^{2}\right)$$

where $N(\mu_{i},\upsilon_{i}^{2})(i=1,2)$ denotes the Gaussian distributions with mean values $\mu_{i}$ and variances $\sigma_{i}^{2}$, and the θ is the mixture coefficient. In this simulation, the mean values $\mu_{1}$ and $\mu_{2}$ both are set at zero, the variances $\sigma_{1}^{2}$ and $\sigma_{2}^{2}$ are set at 1 and 80 respectively, and the mixture coefficient θ is set at 0.25. The overall performance of all algorithms in standard IEEE 14-bus and IEEE 30-bus test system are shown in Figure 2 and Figure 3, respectively. One can observe that the performance of EKF, UKF and A-EKF is significantly worse under the Gaussian mixture measurement noise environment, while the MCC-EKF has good performance because of it is insensitive to the non-Gaussian noise. Moreover, we see that the performance of the proposed AMCC-EKF is better than the original MCC-EKF algorithm. In addition, the average overall performance of all algorithms in standard IEEE 14-bus and 30-bus systems are given in Table 2 and Table 3, respectively.

**Figure 4:**
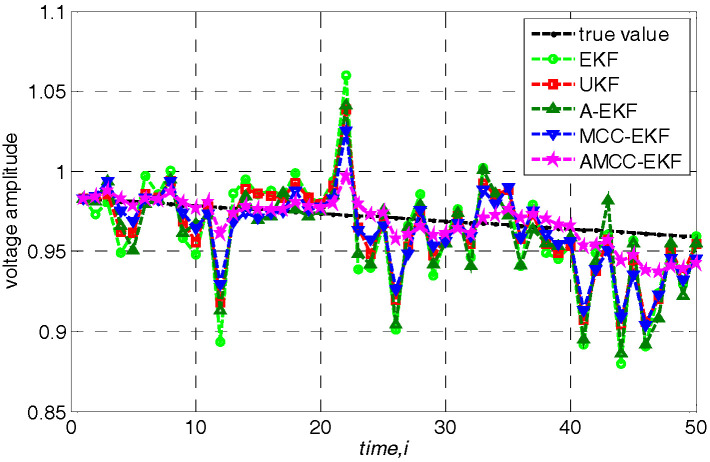
The true voltage amplitude of no.3 bus in IEEE 30-bus test system.

**Figure 5:**
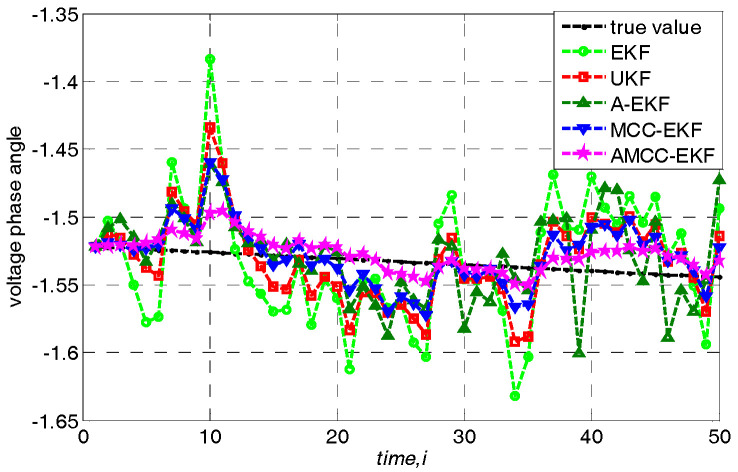
The voltage angle of no.3 bus in IEEE 30-bus test system.

In addition, we further analyze the tracking performance of the algorithm from the perspective of accurately estimating the voltage amplitude and phase angle of bus at each time. Specifically, loads are changed following a linear trend of 10%. The true voltage amplitude and voltage angle of no.3 bus in IEEE 30-bus test system and the estimated values of each algorithms are shown in Figure 4 and Figure 5, respectively. We know that the estimated value of the proposed AMCC-EKF algorithm is close to the true value than other algorithms.

This result proves that the AMCC-EKF has both a higher accuracy and filtering capacities than the corresponding EKF, UKF, A-EKF and MCC-EKF algorithm under the Gaussian mixture measurement noise environment.

### 5.3. Case 3: Laplace and Gaussian Mixture Measurement Noise Environment

In this case, we test the universality of the proposed algorithm under the mixed Gaussian and Laplace noises environment. The noise model can be represented as

**Figure 6:**
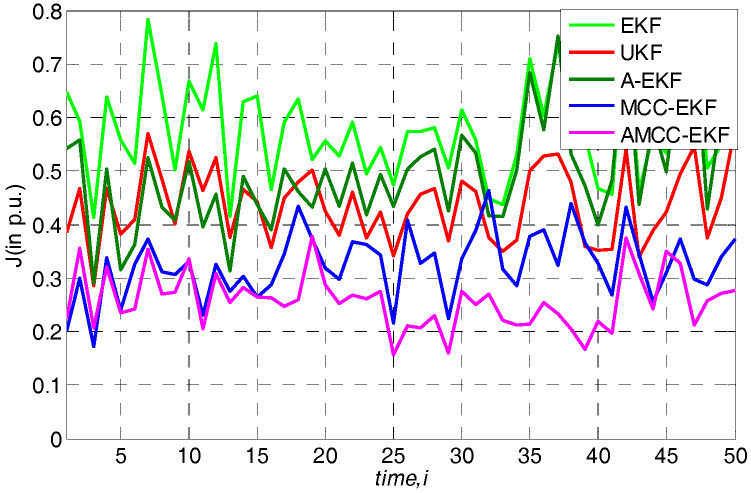
The overall performance of all algorithms in standard IEEE 14-bus under Laplace and Gaussian mixture measurement noise environment.

**Figure 7:**
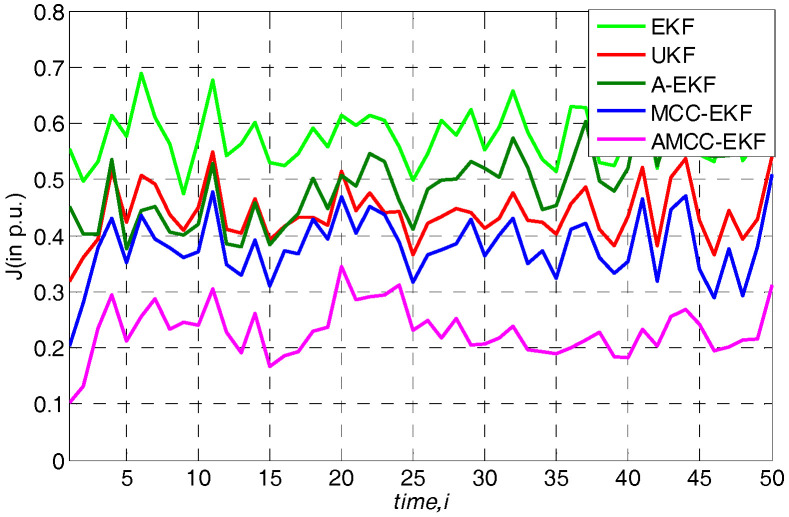
The overall performance of all algorithms in standard IEEE 30-bus under Laplace and Gaussian mixture measurement noise environment.

(74) v(n)=(1−a(n))A(n)+a(n)B(n)

where a(n) is an independent and identically distributed binary process with an occurrence probability 0≤c≤1. In this simulation, c is set at 0.5, A(n) is a noise obey the Laplace distribution with zero-mean and unit variance, and B(n) denotes another noise process with Gaussian distribution with zero-mean and variance 0.55. The noise processes A(n) and B(n) are mutually independent and they are both independent of a(n). The obtained overall performance of all algorithms in IEEE 14-bus and IEEE 30-bus test system are displayed in Figure 6 and Figure 7, respectively. One can see that the EKF, UKF and A-EKF still show worse performance in presence of the Laplace and Gaussian mixture noise. In addition, we can obtain the same conclusion with the Section 5.2 that the MCC based methods (MCC-EKF and AMCC-EKF) are robustness in this case, and the proposed AMCC-EKF algorithm can achieve the best performance. The average overall performance of all algorithms in standard IEEE 14-bus and 30-bus system are shown in Table 4 and Table 5, respectively. The results illustrate the outstanding properties of the AMCC-EKF for SE again.

**Figure 8:**
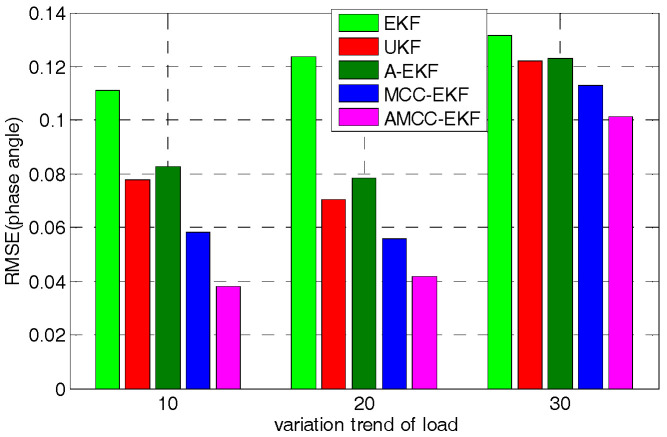
The mean absolute error of voltage angle of no.3 bus in IEEE 30-bus in different variation trend of load.

**Figure 9:**
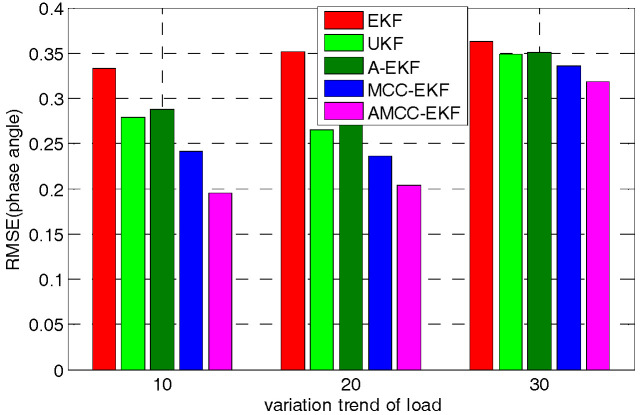
The root mean square error of voltage angle of no.3 bus in IEEE 30-bus in different variation trend of load.

Furthermore, to test the effect of load change on the proposed algorithm, we changed the linear variation trend of load and consider 10%, 20%, 30%, respectively. The mean absolute error and root mean square error of voltage angle of no.3 bus in IEEE 30-bus in different variation trend of load are shown in Figure 8 and Figure 9, respectively. It can be seen from the bar chart that although the error of the proposed algorithm increases with the linear variation trend of the load, the error of the proposed algorithm is minimal compared with other methods.

### 5.4. Case 4: the Nonlinear Variation of Loads

**Figure 10:**
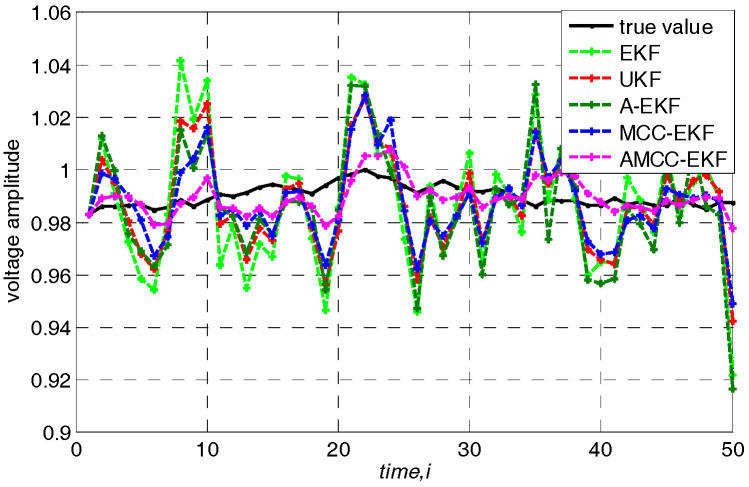
The true value of voltage amplitude of no.3 bus in IEEE 30-bus test system and estimated value of other algorithms when the loads change follows a random fluctuation.

**Figure 11:**
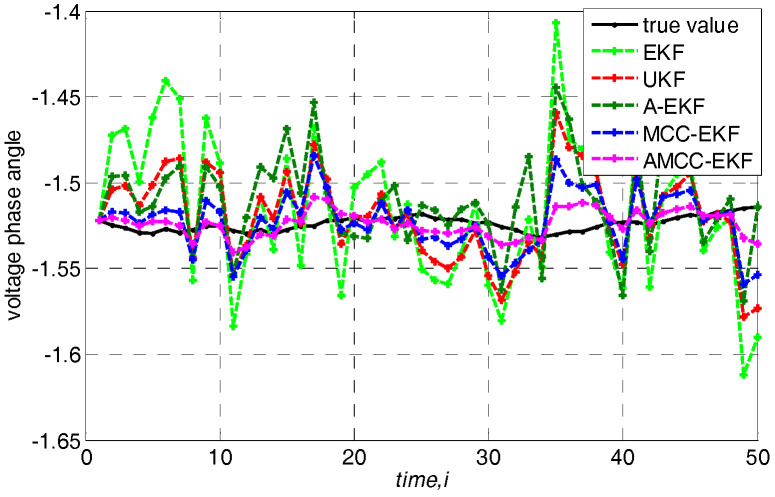
The true value of voltage angle of no.3 bus in IEEE 30-bus test system and estimated value of other algorithms when the loads change follows a random fluctuation.

Now, from the analysis above, we consider this case that the loads change follows a random fluctuation of 5%. The other settings are the same as those of Case 2. The true value of voltage amplitude and angle of no.3 bus in IEEE 30-bus test system and estimated value of other algorithms are shown in Figure 10 and Figure 11, respectively. It can be seen from the figure that even in the case of nonlinear variation of loads, the estimated value of the proposed AMCC-EKF algorithm is closest to the true state value.

To further demonstrate the superiority of the algorithm numerically, the MAE and RMSE of voltage amplitude of no.3 bus in IEEE 30-bus are shown in Table 6, and the results of the proposed AMCC-EKF algorithm is better than other algorithms.

### 5.5. Case 5: in Presence of Outliers

**Figure 12:**
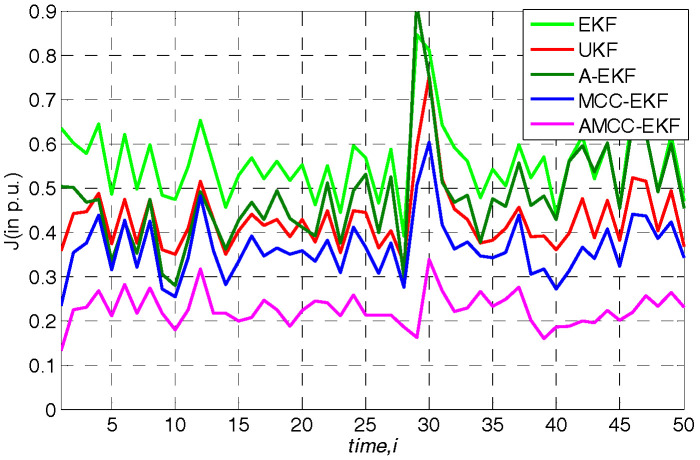
The overall performance of all algorithms in standard IEEE 30-bus in presence of outliers.

Now, measurement in the presence of outliers at sample time 30 is considered in this case and assuming that no measures are taken to verify and identify the bad data under Gaussian mixture measurement noise environment. In this case, four state estimation algorithms are adopted for simulation, resulting in the overall performance index changes of state estimation filter as shown in Figure 12. Firstly, we know that all algorithms are affected by outliers. In the presence of outliers, the filtering performance index will increase and the estimation accuracy will decrease. Secondly, the EKF, UKF, A-EKF and MCC-EKF algorithms are greatly affected by bad data at sample time 30. Although the proposed AMCC-EKF algorithm is also affected to some extent, but the filtering average performance index still fluctuates below 0.33, and the filtering estimation value is relatively correct. The average overall performance of all algorithms in standard IEEE 30-bus are given in Table 7.

## 6. Conclusions

In this paper, a novel AMCC-EKF algorithm is developed to address the power system state estimation problem, and its effectiveness and robustness are verified by some scenarios under non-Gaussian noise environments. First, the actual power system is very susceptible to non-Gaussian noise and the MCC-EKF is employed to design a robust state estimation approach at first. Second, it is difficult to calculate the noise statistics in the most actual situation, and hence the adaptive MCC-EKF (AMCC-EKF) is proposed by introducing the adaptive mechanism into the MCC-EKF algorithm to continuously update the covariance matrix to improve the accuracy of the estimation results. We perform experiments on the IEEE 14 and 30 bus systems to test the performance of the proposed AMCC-EKF method, and the simulation results demonstrate that the filtering performance and estimation accuracy of the AMCC-EKF algorithm for state estimation is better than EKF, UKF, A-EKF and MCC-EKF methods and the estimation results are relatively stable in the presence of outliers.

## Figures and Tables

**Table 1:** The average overall performance of all algorithms in standard IEEE 30-bus.

	EKF	UKF	A-EKF	MCC-EKF	AMCC-EKF
Index J (p.u.)	0.39	0.29	0.31	0.25	0.16

**Table 2:** The average overall performance of all algorithms in standard IEEE 14-bus under Gaussian mixture measurement noise environment.

	EKF	UKF	A-EKF	MCC-EKF	AMCC-EKF
Index J (p.u.)	0.53	0.41	0.49	0.33	0.23

**Table 3:** The average overall performance of all algorithms in standard IEEE 30-bus under Gaussian mixture measurement noise environment.

	EKF	UKF	A-EKF	MCC-EKF	AMCC-EKF
Index J (p.u.)	0.55	0.41	0.48	0.32	0.24

**Table 4:** The average overall performance of all algorithms in standard IEEE 30-bus under Laplace and Gaussian mixture measurement noise environment.

	EKF	UKF	A-EKF	MCC-EKF	AMCC-EKF
Index J (p.u.)	0.65	0.48	0.53	0.36	0.28

**Table 5:** The average overall performance of all algorithms in standard IEEE 30-bus under Laplace and Gaussian mixture measurement noise environment.

	EKF	UKF	A-EKF	MCC-EKF	AMCC-EKF
Index J (p.u.)	0.59	0.52	0.46	0.38	0.23

**Table 6:** The MAE and RMSE of voltage amplitude of no.3 bus in IEEE 30-bus.

	EKF	UKF	A-EKF	MCC-EKF	AMCC-EKF
MAE	0.06	0.05	0.05	0.03	0.01
RMSE	0.25	0.21	0.23	0.18	0.12

**Table 7:** The average overall performance of all algorithms in standard IEEE 30-bus in presence of outliers.

	EKF	UKF	A-EKF	MCC-EKF	AMCC-EKF
Index J (p.u.)	0.56	0.46	0.48	0.36	0.24
